# Reevaluating scorpion ecomorphs using a naïve approach

**DOI:** 10.1186/s12862-022-01968-0

**Published:** 2022-02-14

**Authors:** Pedro Coelho, Antigoni Kaliontzopoulou, Pedro Sousa, Mark Stockmann, Arie van der Meijden

**Affiliations:** 1 CIBIO, Centro de Investigação em Biodiversidade e Recursos Genéticos, InBIO Laboratório Associado, Campus de Vairão, Universidade do Porto, 4485-661 Vairão, Portugal; 2 Departamento de Biologia, Faculdade de Ciências, Universidade do Porto, 4099-002 Porto, Portugal; 3 BIOPOLIS Program in Genomics, Biodiversity and Land Planning, CIBIO, Campus de Vairão, 4485-661 Vairão, Portugal; 4 Department of Evolutionary Biology, Ecology and Environmental Sciences, and Biodiversity Research Institute (IRBio), Universitat de Barcelona, 08028 Barcelona, Catalonia Spain; 5 Im Hoek 20, 48477 Hörstel-Riesenbeck, Germany

**Keywords:** Ecomorphological diversity, Scorpions, Microhabitat, Ecomorphology

## Abstract

**Background:**

Ecomorphs create the opportunity to investigate ecological adaptation because they encompass organisms that evolved characteristic morphologies under similar ecological demands. For over 50 years, scorpions have been empirically assigned to ecomorphs based on the characteristic morphologies that rock, sand, vegetation, underground, and surface dwellers assume. This study aims to independently test the existence of scorpion ecomorphs by quantifying the association between their morphology and ecology across 61 species, representing 14 families of the Scorpiones order.

**Results:**

Without a priori categorization of species into ecomorphs, we identified four groups based on microhabitat descriptors, which reflect how scorpion ecospace is clustered. Moreover, these microhabitat groups, i.e., ecotypes, have significantly divergent morphologies; therefore, they represent ecomorphs. These ecomorphs largely correspond with the ones previously described in the literature. Therefore, we retained the names Lithophilous, Psammophilous, and Pelophilous, and proposed the name Phytophilous for vegetation dwellers. Finally, we sought to map the morphology-ecology association in scorpions and found that the morphological regions most tightly associated with ecology are at the extremities. Moreover, the major trend in ecomorphological covariation is that longer walking legs and relatively slender pedipalps (pincers) are associated with sandy microhabitats, while the inverse morphological proportions are associated with rocky microhabitats.

**Conclusions:**

Scorpion ecomorphs are validated in a naïve approach, from ecological descriptors and whole body anatomy. This places them on a more solid quantitative footing for future studies of ecological adaptation in scorpions. Our results verify most of the previously defined ecomorphotypes and could be used as a current practice to understand the adaptive significance of ecological morphology.

**Supplementary Information:**

The online version contains supplementary material available at 10.1186/s12862-022-01968-0.

## Introduction

Ecological adaptation is the process whereby organisms respond to environmental change by modifying their phenotype. Selection triggers adaptation to new environments and ecological niches, resulting in divergence. If the adaptations include changes in external morphology, we can observe this type of divergence in the form of recurrent morphologies—ecomorphs. Ecomorphs are groups of organisms that share a set of morphological traits associated with specific ecological requirements. Selection for these characteristic morphologies occurs in association with enhancing the ecologically relevant performance that morphology permits, also modulated by associated behaviors [1–5]. Striking examples of ecomorphs are predominantly known from recent adaptive radiations, such as cichlid fish [6] and *Anolis* lizards [7, 8]. Although with fewer examples, ecomorphs originating from older radiations are also known, such as beetles and salamanders [9, 10].

Scorpions are particularly suitable for studying ecomorphology because they have a functionally compartmentalized anatomy. As such, the functions and behaviors related to intraspecific competition, prey incapacitation, defense against predators, feeding, sensing, and sexual courtship are carried out by four anatomical regions: the pedipalps, prosoma, metasoma, and telson [11–16]. In other organisms, these functions may all be carried out using a single anatomical region, such as the head in lizards [17], while in scorpions, some functions are limited to one or more of these anatomical regions. For instance, prey capture and manipulation are restricted to the pedipalps, while prey incapacitation and defense often include the telson. Consequently, in scorpions, differences in these anatomical regions are more easily assigned to different functional demands.

Another reason makes scorpions an interesting group for studying the ecological adaptation of their morphology: they have long been assigned to ecomorphs [18–21]. These ecomorphs are considered to be associated with microhabitat use, specifically the substrate that scorpions use. The five major scorpion ecomorphs previously identified are vegetation dwellers (corticolous) [22, 23], rock climbers (lithophilous) [24, 25], hard soil fossorials (pelophilous) [23, 26], sand fossorials (psammophilous) [27, 28], and ground surface vagrants (lapidicolous) [20, 29]. Among these, there are substrate specialists and substrate generalists, i.e., habitat stenotopes and habitat eurytopes, sensu [29]. Psammophiles and lithophiles are habitat stenotopes because the extensive morphological adaptations for their preferred substrates—loose sand and rocks—make such species unfit to occupy other substrates. Lapidicolous, corticolous and pelophilous ecomorphs are eurytopic [23], with lapidicolous being the least specialized group. Other scorpiologists labeled ecomorphs differently, yet this reflects a terminological difference rather than a conceptual one [23, 30]. Some of the described morphological characteristics of these ecomorphs are considered functional adaptations. Psammophilous scorpions have longer setae and claws on their feet that may spread their mass over a larger area and reduce energy wasted on displacing loose sand during locomotion. Also, the streamlined metasoma and telson are suggested to increase sand burrowing efficiency [28] and aid in escape when animals become buried in the sand [20]. In pelophilous species, the robust, crab-like chelae are proposed to aid in burrowing, although not all species with large chelae use them for this function [29]. In addition, a lifetime spent inside a burrow is thought to reduce telson size due to its diminished use [20]. Lithophilous species are associated with dorsoventral compression and often elongation of the pedipalps, legs, and metasoma. This trend, however, seems more evident in rock crevice dwellers than in other rock microhabitats [29]. Corticolous species are also associated with some elongation of the pedipalps and legs. Other trends in corticolous scorpions seem to be more phylogenetically specific, such as the elongation of the metasoma in buthids and a general dorsoventral compression of the body in non-buthids [29]. Nonetheless, these examples are largely empirical, featuring descriptions of both regional scorpiofauna or clade-specific groups. No global study has tested whether scorpions inhabiting different habitats and continents can be grouped together based on their ecological preferences; and whether such groups also exhibit morphological coherence across the scorpion phylogeny. In sum, there is no overall data-based validation that scorpion ecomorphs exist.

Confirming that scorpion ecomorphs exist involves the simultaneous analysis of morphological and ecological traits in a phylogenetic comparative framework. Our main questions are: Can scorpions be grouped based on microhabitat preference? If so, are these groups morphologically distinct? Do these groups correspond to currently recognized ecomorphs? Which morphological traits co-vary more strongly with ecology? Given the recurrent association of characteristic pedipalp and “tail” (metasoma and telson) shapes for each ecomorph, we hypothesized that the extremities are the ecologically most important anatomical parts.

## Materials and methods

### Sampling and phylogeny

To explore the morphological diversity (phenotype) across the Scorpiones order, we made morphological measurements on 61 species. We measured one specimen per species, spanning approximately 70% of extant families (14 of 20), five continents (North and South America, n = 20; Eurasia, n = 15; Africa, n = 24; Oceania, n = 2). The Scorpiones families are represented by the Chaerilidae (n = 1), Buthidae (n = 23), Iuridae (n = 1), Bothriuridae (n = 3), Chactidae (n = 5), Scorpiopiidae (n = 3), Euscorpiidae (n = 1), Troglotayosicidae (n = 1), Caraboctonidae (n = 2), Hormuridae (n = 8), Urodacidae (n = 1), Diplocentridae (n = 2), Scorpionidae (n = 5) and the Vaejovidae (n = 5). The ecomorphs included are those referenced in the literature as psammophilous (e.g., *Apistobuthus pterygocercus* and *Vejovoidus longuiunguis*), lithophilous (e.g., *Hadogenes paucidens* and *Iurus dufoureius*), pelophilous (e.g., *Pandinoides cavimanus* and *Odontobuthus doriae*), corticolous (e.g., *Tityus trinitatis* and *Opisthacanthus asper*) and lapidicolous scorpions (e.g., *Buthus ibericus* and *Bothriurus coriaceus*). Specimens were selected from the scorpion collection at CIBIO in Vila do Conde, Portugal, the RMNH in Leiden, the Netherlands, and the MNHN in Paris, France.

We used recent transcriptome-based phylogenies of the Scorpiones order for our comparative analyses [31, 32]. From there, we drew an ultrametric tree with the same topology as the higher-level classifications. The interfamilial relationships are congruent in both publications, except for the position of the Vaejovidae, which was revised in [32] and adopted in this study. Species that were not present in either phylogenetic analysis were treated in one of two ways: species with members of the same genus represented in the phylogenies were placed at the position of the genus; species without genus representation were placed sister to the corresponding family clade [33, 34]. In all cases, unclear relationships between species were represented with polytomies. Branch lengths were adjusted to the tree topology, calculated using Grafen’s method [35]. We also performed the same analysis using the latest transcriptome-based phylogeny of Santibáñez-López et al. [36].

### Morphological measurements

We aimed for an unbiased sampling of scorpion anatomy by measuring all anatomical regions of the scorpion body. Specifically, we did not restrict our sampling to known eco-functional traits. We used digital calipers (Absolute IP67, Mitutoyo Inc., Kawasaki, Japan) to measure 70 lengths from six anatomical regions, namely in the prosoma, mesosoma, metasoma, telson, walking legs, and pedipalps, to the nearest 0.01 mm, following Stahnke [37]. Our measurements did not include structures such as the chelicerae, carinae, tarsi, or the setal hairs. The measured species exhibit considerable size variation, and our sampling represents total body lengths from 22.1 mm in *Microbuthus* sp. to 158.05 mm in *Ha. granulatus*. To reduce the number of variables for statistical analysis, we summed trochanter and femur lengths into a “proximal leg” part and the patella, tibia, and metatarsus lengths into a “distal leg” part. This division corresponds to biomechanically functional units of the leg: on one side, the distal muscles, responsible for pretarsal (“foot”) movement occupy all distal segments until and including the patella; on the other side, most of the leg motion occurs around the femoropatellar joint [38]. The lengths of the five metasoma segments were added together, while their heights and widths were averaged. Pedipalp measurements represent averages between left and right pedipalps. For details about the measurements (description, abbreviation, and illustration), see Additional file 1: Fig. S1 and Additional file 4: Table S1. The final morphological dataset consisted of 36 morphological variables, which were log-transformed before further analysis (Additional file 5: Table S2). In general, scorpions are not characterized by strong sexual dimorphism, so we did not differentiate specimens by sex. However, in those species with more substantial sexual dimorphism, males and females may have subtly different ecological roles [39].

Unlike in, e.g., herpetology, no consensually accepted single linear measurement corresponds well with overall body size in scorpions [40]. Therefore, an isometric body size (IsoSize) was calculated by projecting the 36 linear measurements on an isometric vector. We then calculated each linear measurement's regression residuals using IsoSize to obtain size-corrected traits, following [41]. Last, we calculated the degree of phylogenetic signal present in the morphological variables given the phylogeny using the function *physignal* of the of R package GEOMORPH. *physignal* provides a mathematical generalization of the Kappa statistic [42] appropriate for highly multivariate data [43].

### Ecological data and microhabitat clustering

Since the conceptualization of the five scorpion ecomorphs, not all species have been assigned to one. For example, less than 50% of the taxa selected here are unambiguously assigned to an ecomorph in the literature. In cases where an ecomorph assignment can be found in the literature, the assignment is often made based on the morphological habitus of the specimens, risking circular reasoning. To overcome these limitations, we chose to forego the assignments to classical ecomorphs entirely in our analysis. Instead, we retrieved descriptors of microhabitat use from the literature. We selected descriptors referring to substrates, their arrangement, and the activities scorpions perform in them. Those most frequently encountered in the literature were used to record presence (= 1) vs. unreferenced presence (= 0) for the following 12 parameters: “compact soil”, “loose sand”, “rock surface”, “leaf-litter”, “under rocks”, “under vegetation”, “dug burrow”, “passive shelter”, “climb bushes”, “climb trees”, “rock crevices” and “hanging upside down”. Each species was assigned one or more microhabitat uses (see Additional file 5: Table S2).

We grouped species with similar microhabitat uses together into clusters. To achieve this, we used the matrix of 12 microhabitat traits in the following three steps. First, we calculated Jaccard distances between pairs of species based on all microhabitat descriptors together, using the function *vegdist* of R package vegan [44]. Secondly, using the obtained distance matrix, we selected the number of habitat clusters based on the Bayesian Information Criterion (BIC), using the function *Mclust* of R package mclust [45] (Additional file 2: Fig. S2). We selected the number of clusters corresponding to the first k preceding a plateau in BIC values. The number of clusters, four (see “Results”), was used to compute a k-means clustering. The distance matrix was used as input for a Principal coordinate analysis (PCoA), resulting in 26 axes. Lastly, we reviewed the ecological composition of each microhabitat cluster by calculating two matrix correlations (Spearman’s as well as Pearson’s ρ): (1) between the microhabitat traits and the PCoA scores to obtain PCoA-to-microhabitat correspondence; (2) between the resulting matrix from 1) and the k-means cluster centers to obtain microhabitat-to-cluster correspondence. Cluster terminology reproduces the different associations of each cluster to the microhabitat traits.

To visualize the scorpion ecospace, we performed multiple correspondence analyses (MCA) using function *MCA* of R package FactoMineR  [46]. MCA uses the PCoA scores of microhabitat traits to plot barycenter points of categories (n-individual mean scores) and barycenter points of individuals (n-category mean scores) simultaneously; for visual clarity, however, only the latter were plotted.

### Ecology-morphology associations

We searched for strongly eco-covarying traits by exploring which morphological traits co-vary most with ecology across all species. To this end, we applied a phylogenetic Partial Least Squares (PLS, using the phylogeny shown in Fig. 2) to the multivariate sets of ecological and morphological variables using the function *phylo.integration* [47–49] of R package GEOMORPH [50]. Here, multivariate ecology consisted of 26 microhabitat traits (PCoA axes), and multivariate morphology consisted of 36 size-corrected morphological traits. Permutations with 10,000 cycles were used to test for significance of the multivariate correlation between vectors of morphology and ecology. The resulting matrix of morphological traits with maximized ecological covariance is hereafter referred to as the matrix of eco-projected morphology.

### Ecomorphological distinctiveness between microhabitat clusters

To corroborate the existence of ecomorphs in scorpions, we examined whether microhabitat clusters exhibit distinctive phenotypes as represented by ecologically correlated morphology. For this purpose, we performed a MANOVA with the matrix of eco-projected morphology as the dependent variable and microhabitat cluster as a predictor while accounting for phylogenetic autocorrelation using generalized least squares (GLS). To test for significance, we used randomization of residuals over 10,000 permutations, as implemented in the function *lm.rrpp* of the RRPP R package [51, 52]. Then, to identify which microhabitat clusters differed significantly, we employed distance-based testing of pairwise differences between microhabitat cluster means, as implemented in the function *pairwise* of RRPP [53, 54]. For illustration, we plotted group means rotated to their principal components and with 95% confidence ellipses around them, using the plotting tools of the RRPP R package.

## Results

### Microhabitat clusters as a proxy for ecomorphs

**Fig. 1:**
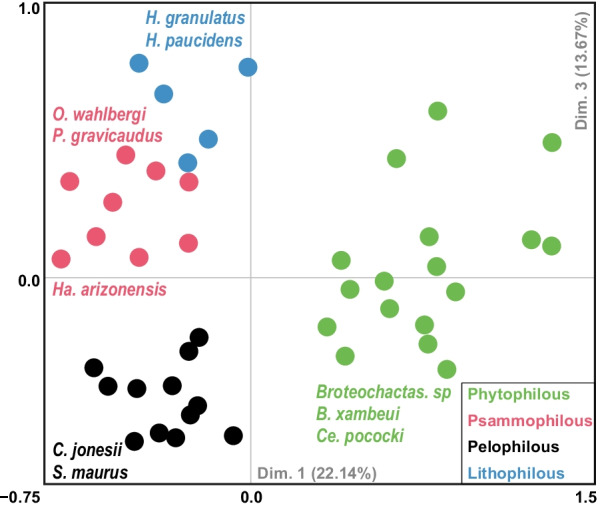
PCoA map of scorpion ecospace. The ecospace is depicted by species (dots) colored according to ecomorph affiliation. Dots are colored based on the four clusters obtained with the R package mclust. For illustration purposes, and because it explains approximately the same variation as the second PCoA dimension, we plotted the third PCoA dimension in the vertical axis. Moreover, only species with a cos^2^ correlation higher than 0.70 with both dimensions for the pelophilous and phytophilous ecomorphs, and a cos^2^ correlation higher than 0.45 for the psammophilous and lithophilous ecomorphs are labeled. Percentage values refer to the variation explained by each axis. See Additional file 3: Fig. S3 for contributions of microhabitat variables to axis composition

Based on the BIC, the ecological data supports the existence of four clusters (Additional file 2: Fig. S2). In terms of ecological composition, the microhabitat clusters show different associations with microhabitat traits (Table 1, Additional file 3: Fig. S3): Cluster 1 is primarily dominated by species occurring on loose sand and digging burrows; Cluster 2 mainly contains species found under vegetation, in leaf litter and using passive shelters; Cluster 3 is mostly comprised of species found in compact soil and digging burrows and Cluster 4 has a higher contribution of scorpions exploring both the crevices and the surface of rocks (Fig. 1, Table 1). It is worth noting that scorpions that rest hanging upside down are closest to cluster 4, while those climbing trees and bushes only positively correlate with cluster 2. Therefore, we named these clusters “Psammophilous”, “Phytophilous”, “Pelophilous” and “Lithophilous”, respectively, following the most common ecomorph terminology (Fig. 1, Table 1, Additional file 5: Table S2). Dimensions 1 and 2 of the MCA together capture about 40% of the microhabitat variation. Across this ecospace, the microhabitat clustering produced ecomorph groups with reduced overlap (Fig. 1).

**Table 1:** Microhabitat cluster affiliation with microhabitat traits

K-means clusters	Cluster 1	Cluster 2	Cluster 3	Cluster 4
Microhabitat traits	Psammophilous	Phytophilous	Pelophilous	Lithophilous
Under vegetation	− 0.42	0.82	− 0.53	− 0.26
Leaf litter	− 0.39	0.73	− 0.53	− 0.08
Passive shelter	− 0.41	0.56	− 0.26	− 0.06
Climb trees	− 0.13	0.32	− 0.29	− 0.02
Under rocks	− 0.61	0.31	0.25	0.08
Climb bushes	− 0.06	0.24	− 0.24	− 0.05
Hanging upside down	− 0.07	− 0.05	0.08	0.13
Rock crevices	− 0.23	− 0.18	0.08	0.80
Compact soil	− 0.21	− 0.18	0.69	− 0.31
Rock surface	− 0.19	− 0.24	0.14	0.77
Loose sand	0.83	− 0.40	− 0.23	− 0.36
Dug burrow	0.54	− 0.66	0.47	− 0.29

Values correspond to Pearson’s ρ correlation, on which the terminology adopted for the ecomorphs was based

**Fig. 2:**
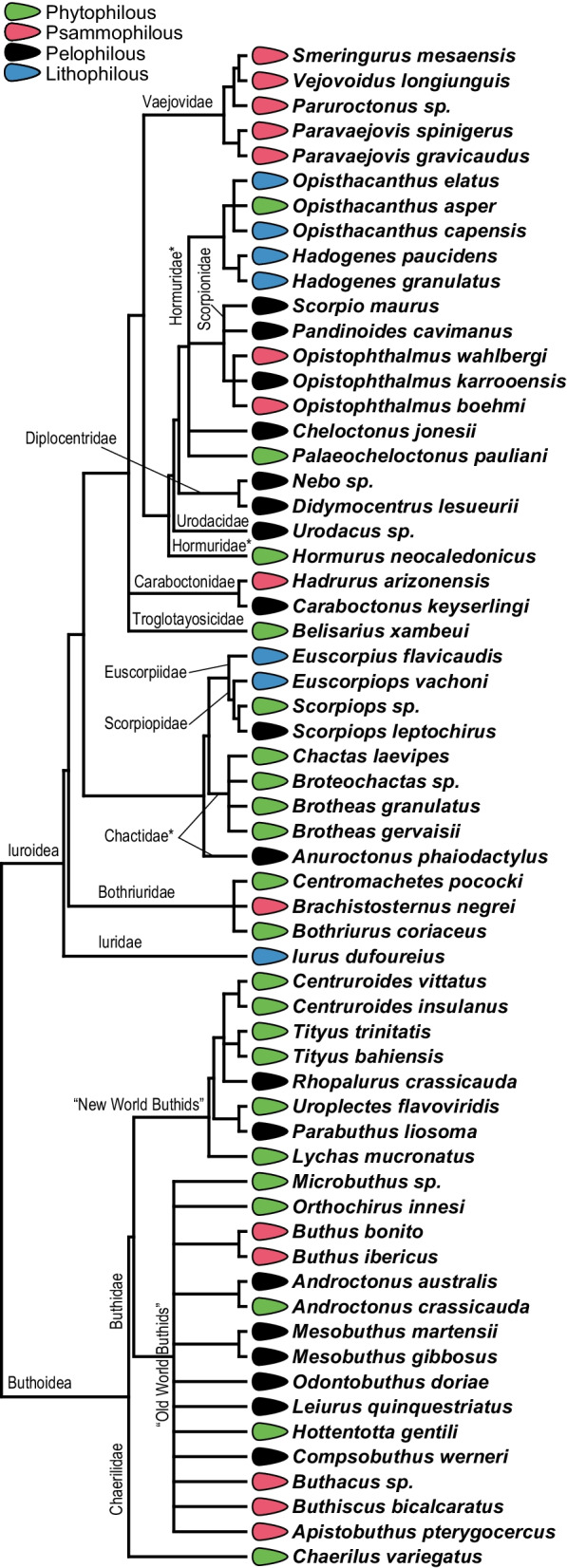
Phylogenetic relationships of the scorpion species included in this study. Names of the families and superfamilies are indicated. Paraphyletic families are indicated with an asterisk. Colors correspond to microhabitat ecomorph affiliation. Tree topology is based on Santibáñez-López et al. (2019) [32] and Sharma et al. (2015) [31]

Our phylogenetic tree of scorpions reveals that most scorpion families contain representatives of at least two ecomorphs (Fig. 2). In this study, the exceptions are the Vaejovidae and Diplocentridae families, as we sampled only Psammophilous and Pelophilous species respectively from, despite other ecomorphs existing in these families. This is an artifact of the limited sampling of these families, especially of the highly speciose and diverse Vaejovidae. At the superfamily level, the Iuroidea has representatives of all ecomorphs, while the Buthoidea lacks the Lithophilous ecomorph in our sampling. As a reminder, the absence of the Lithophilous ecomorph does not imply that Buthoidea scorpions are not associated with rocks. Although more prevalent in Lithophilous species, rock surfaces are also part of the Pelophilous niche (Table 1).

The phylogenetic history of scorpions has a significant influence in their morphology. As such, the metasoma average width and height are the traits with the highest phylogenetic signal among the sampled taxa (Additional file 8: Table S5).

### Ecology-morphology associations

**Fig. 3:**
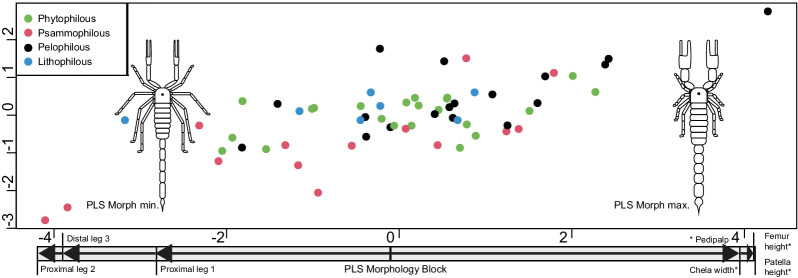
Phylogenetic PLS between scorpion ecology (Y-axis) and morphology (X-axis). Morphological traits with the highest covariation with ecology are labeled on the X-axis. Scorpion illustrations correspond to sand burrowing microhabitats (on the left, minimum extreme of the morphology PLS vector) and microhabitats found on and under rocks (on the right, maximum extreme of the morphology PLS vector). Colors correspond to microhabitat ecomorph affiliation

Examination of the multivariate association between morphology and ecology yielded significantly correlated PLS vectors (r = 0.698, z = 3.228, p < 0.001) (Fig. 3). Vectors of ecology and morphology revealed that scorpions living under and on rock surfaces are associated with pedipalps with (dorsoventrally) higher femurs, wider patellas, and both wider and higher chelae. They are also associated with leg pairs 1 and 2 having shorter proximal parts and leg pairs 2 and 3 having shorter distal parts. Conversely, scorpions living in loose sand and digging burrows have inverse anatomical proportions (Fig. 3). As a reminder, we attributed the term eco-projected morphology to the morphological dataset resulting from this PLS; in it, the morphological traits with the highest ecological correlation, i.e., strongly eco-covarying traits, are located in the walking legs and the pedipalps of scorpions (Fig. 3).

### Ecomorphological distinctiveness between ecomorphs

**Fig. 4:**
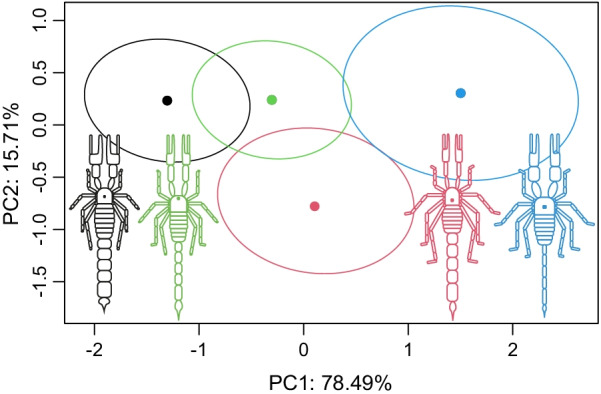
Ecomorph means (filled dots) rotated to their principal components, and 95% confidence ellipses around them. Illustrations approximate the mean morphologies of each ecomorph, and colors correspond to ecomorph affiliation

MANOVA comparisons show significant differences in eco-projected morphology across the four microhabitat clusters (Table 2). Consequently, microhabitat clusters are hereafter called ecomorphs; posthoc tests show which ecomorphs are significantly different (Additional file 6: Table S3). Mean morphologies and respective confidence ellipses, estimated from the MANOVA, confirm the reduced overlap between the different ecomorphs (Fig. 4). The illustrations of four scorpions approximate the mean morphologies of each ecomorph.

**Table 2:** MANOVA comparisons of eco-projected morphology across microhabitat clusters

MANOVA	Df	SS	MS	Rsq	F	Z	P
hab	3	646.4	215.475	0.121	2.603	2.484	**0.006**
Residuals	57	4718.1	82.773	0.880			
Total	60	5364.5					

Significance testing is based on 10,000 cycles of residual permutations. Degrees of freedom (Df), Sums of Squares (SS), R squared (R^2^), F value (F), effect size (Z), and corresponding p-value. Significant effects at an alpha of 0.05 are marked in bold font

## Discussion

In this paper, we independently corroborate the existence of scorpion ecomorphs, and show that they are largely congruent with, but not identical to those previously defined. Moreover, we found that some parts of the scorpion body vary more strongly with ecology than others, yet not those body parts we expected.

### Microhabitat clusters are ecomorphs

To start with a clean slate and avoid circular reasoning, we summarized microhabitat use in clusters rather than assigning species to previously defined ecomorphs. The number of clusters recovered in this study, four, differs from the five ecomorphs previous literature would have assigned our sampling to [29, 55]. We validated that our microhabitat clusters represent ecomorphs by showing that morphological variation is significantly different between them (Table 2). This validation accounted for two factors that mask anatomical similarity: body size and phylogeny.

Our results corroborate most ecomorphs already defined in bibliography, and thus we generally use the same names for the sake of continuity. However, we propose changing the designation “Corticolous” to “Phytophilous” as we feel that this name is more apt given the microhabitat descriptors associated with that ecomorph: “under vegetation”, “leaf-litter” and “passive shelter”. Contrary to the five ecomorphs from literature, we found our ecospace was best segmented into four clusters, where adding a fifth cluster did not improve the model. Our classification of scorpion ecomorphs, therefore, lacks the ecologically “catch-all” Lapidicolous ecomorph. The Phytophilous ecomorph, positively associated with six different microhabitats, contains the most generalist species, followed by the Pelophilous ecomorph. This trend corroborates Prendini’s [29] hypothesis that vegetation climbers and hard soil fossorials are microhabitat generalists. The Psammophilous and Lithophilous ecomorphs remain as habitat specialists, with strong correlations with a maximum of two microhabitats (Fig. 1, Table 1).

We acknowledge that these results, particularly on the number of clusters recovered and the species they consist of, are dependent on the choice and number of sampled taxa, and the prevalent phylogenetic hypothesis. After we completed the statistical analysis, a new phylogenetic hypothesis was published, placing *Hadrurus* outside the Caraboctonidae, among other topological changes [36]. The overdue molecular phylogenetic reappraisal of the phylogenetic relationships among scorpion taxa has recently accelerated, making this latest revision one of many to come before the high-level phylogenetic hypothesis of scorpions can be expected to converge to a stable situation. Performing our analysis again using this latest transcriptome-base phylogeny confirms the validity of scorpion ecomorphs since the same microhabitat clusters are morphologically distinct, although to a lower magnitude of correlation. Moreover, longer walking legs remain significantly associated with digging burrows and microhabitats with loose sand, but also under rocks (Additional file 9: Table S6). Under this slightly different phylogenetic hypothesis another body section gains more ecological relevance: the metasoma and telson. Wider, higher and longer metasomas and telsons are associated with compact soil and rock surface microhabitats but also with climbing bushes and trees.

Overall, we sampled 61 of the approximately 2.500 species of scorpions described: less than 3%. Although we strove for a broad sampling, families such as the species-rich radiation of the Vaejovidae and the Australo-Papuan radiations of Hormurids, Scorpiopids, and Urodacids, among others, were underrepresented. Moreover, the clustering results may be further influenced by not including representatives of the troglophilous ecomorph, and future studies should attempt to include a sufficient sample of this minor, yet more specialized, ecomorph. Future taxonomical updates will converge towards a more robust picture of the Scorpiones tree of life. We here provide a method that can accommodate virtually any phylogenetic assemblage and topology.

### The extremities are rich in eco-covarying traits

The strongly eco-covarying traits, highlighted in PLS axes (Fig. 3), span two quite different anatomical regions: scorpions with relatively slenderer pedipalps and relatively longer legs in one extreme and scorpions with more robust pedipalps and shorter legs in the other. These shapes are the theoretical limits of morphological variation associated with sand fossorial or rock-dwelling microhabitats, respectively (but not rock crevice microhabitats). In fact, such rock eurytopes seem to be ecologically closer to the semilithophilous ecomorph sensu [29]. These trends, shown by the PLS, are relative. While in absolute terms, e.g., a loose sand burrowing scorpionid (a family of scorpions with typically robust pedipalps) may still have more robust pedipalps than, e.g., a rock-dwelling buthid, it will have slenderer pedipalps relative to other scorpionids (taxonomical relationships are depicted in Fig. 2).

The extremities are of major importance for scorpions to interact with the environment: the walking legs operate on the substrate in locomotion, and the pedipalps are used to navigate, obtain prey, defend and mate [56–58]. Different microhabitats may pose different demands on the functions of these two anatomical modules. Nonetheless, pedipalps and walking legs also share many developmental pathways [59], potentially leaving them less free to vary with ecology. Developmental linkage is more evident for structures along the body axis, as they share many developmental pathways [59, 60]. The ecomorphological paradigm predicts that the various ecomorphological associations uncovered here are likely functional, but this cannot be concluded from correlation alone. Nevertheless, these results can be an informed starting point for experimental validation of the functional value of those traits.

Based on the numerous mentions of the ecological role and morphological distinctiveness that the pedipalps and the “tail” (metasoma and telson) get in descriptions of scorpion ecomorphs, we hypothesized that in our dataset they would co-vary the most with ecology. To our surprise, variation in the metasoma and telson is not as strongly correlated with microhabitat use as the walking legs given our phylogenetic sampling and topology. As mentioned before, it looks like the appendages that directly interact with the substrate (walking legs) or to navigate through the environment (pedipalps), are more strongly associated with ecology. A potential reason for “tail” traits not emerging as part of a general ecomorphological trend in scorpions involves phylogenetic-specific associations. In fact, the phylogenetic signal of the metasoma average height and width are the highest among all the morphological measurements; and the width of the telson vesicle within the ten highest values (Additional file 8: Table S5). Accordingly, when the phylogenetic history of scorpions is not taken into account, the pedipalps and both the metasoma and the telson are the anatomical regions with the strongest association with ecology (Additional file 7: Table S4). Our data shows that morphological variation in the metasoma and telson has a stronger phylogenetic signal than an ecological signal [29] noted that the elongation of the metasoma in corticolous (i.e., phytophilous) scorpions was only verified in the Buthidae family. This observation highlights that morphological variation in the “tail” of scorpions may have a strong phylogenetic signal overall, or even vary by family. The latter may be supported by the gain in ecomorphological relevance by the “tail” in the supplementary analysis that adopts a redefined Caraboctonidae family. Conversely, this may indicate that the ecomorphological importance of walking legs proportions may have been partly obscured by phylogenetic signal.

Because we found several morphological traits associated with ecology, the use of such characters for phylogenetic inference is not advised. For example, there are species placed at the extremes of Fig. 3, such as *Scorpio maurus*, and *Opistophthalmus wahlbergi*, which belong to same family, the Scorpionidae. These species are, therefore, quite divergent in both ecology and morphology. Phylogenetically neutral morphological traits would place these two species closer together than with species from other taxonomic families.

## Conclusions

This study is the first broad quantitative approach to scorpion ecomorphs. We were able to identify associations between ecology and morphology, which transcend taxonomical relatedness. The ecomorphological paradigm predicts that the various associations we uncovered are likely functional, but correlation alone is not conclusive evidence. Nevertheless, these results can be an informed starting point for experimental validation of the functional value of those traits.

## Supplementary Information

**Additional file 1.** Arrows represent the distance measured by calipers. Measurements of the left column are colored according to their anatomical region: pedipalps, prosoma, mesosoma, metasoma, and walking legs).

**Additional file 2.** Model-Based Clustering performance for clustering of samples via the BIC for up to 10 components (i.e. clusters) (BIC = Bayesian Information Criterion). EII - VVI refer to the names of the models used for clustering fitting.

**Additional file 3.** The ecospace is depicted by species (circles) and microhabitat use (triangles) and ecomorph groups (squares) colored according to ecomorph affiliation. The intensity of the colors corresponds to the cos2 correlation with both dimensions. Percentage values refer to the variation explained by each axis. Ecomorph datapoints are supplementary information that does not contribute to axis composition.

**Additional file 4.** Abbreviations of the linear morphological measurements. Name abbreviation, description, and anatomical location of the morphological measurements. The statistical analysis uses a data reduction from 70 to 36 morphological variables.

**Additional file 5.** Microhabitat use and morphological dataset. Absolute values of 36 linear measurements, 12 microhabitat variables, and the ecomorphs resulting from microhabitat clustering.

**Additional file 6.** Pairwise distances between Least Squares Regression (LSR) means calculated by a MANOVA of eco-projected morphology.

**Additional file 7.** Partial Least Squares (PLS) loadings. Loadings of the first Partial Least Squares vector (PLS1) for each multivariate block (morphology and microhabitat) in two scenarios: when phylogeny is taken into account (left columns) and when phylogeny is not taken into account (right columns).

**Additional file 8.** Phylogenetic signal of the morphological measurements. Blomberg's K values of phylogenetic signal and respective test of significance (P value).

**Additional file 9.** Revised Partial Least Squares (PLS) loadings. Loadings of the first PLS (PLS1) for each multivariate block (morphology and microhabitat) corrected for the phylogeny of "Santibáñez-López et al. [36].

## Data Availability

All data generated or analyzed during this study are included in this published article and its Additional information files.
