# Supplementary material for: Reevaluating scorpion ecomorphs using a naïve approach
Source: BMC Ecol Evol. 2022 Feb 14;22:17. doi: 10.1186/s12862-022-01968-0 (PMC8845257; doi:10.1186/s12862-022-01968-0)
Supplement: Supplementary file 1 — Additional file 1. Arrows represent the distance measured by calipers. Measurements of the left column are colored according to their anatomical region: pedipalps, prosoma, mesosoma, metasoma, and walking legs). [file 12862_2022_1968_MOESM1_ESM.pdf]

L Prosoma
L Median eyes to front edge
W Prosoma, midlle
W Prosoma
W Mesosoma 1th terg.
W Mesosoma 4th terg.
L Mesosoma (4th terg.)
W Mesosoma 7th terg.
L Metasoma 1st
L 2nd
L 3rd
L 4th
L 5th
W 1st
W 2nd
W 3rd
W 4th
W 5th
H 1st
H 2st
H 3rd
H 4th
H 5th
L telson
H vesicle
W vesicle
L left leg1 trochanter
L left leg1 femur
L left leg1 patella
L left leg1 tibia
L left leg1 metatarsus
L left leg1 tarsus
L left leg2 trochanter
L left leg2 femur
L left leg2 patella
L left leg2 tibia
L left leg2 metatarsus
L left leg2 tarsus
L left leg3 trochanter
L left leg3 femur
L left leg3 patella
L left leg3 tibia
L left leg3 metatarsus
L left leg3 tarsus
L left leg4 trochanter
L left leg4 femur
L left leg4 patella
L left leg4 tibia
L left leg4 metatarsus
L left leg4 tarsus
L left Pedipalp femur
W left femur
H left femur
L left patella
W left patella
H left patella
L left chela
W left chela
H left chela
L left mov finger
L right Pedipalp femur
W right femur
H right femur
L right patella
W right patella
H right patella
L right chela
W right chela
H right chela
L right mov finger

Supplementary Figure 1: Illustration of the linear morphological measurements across the scorpion body

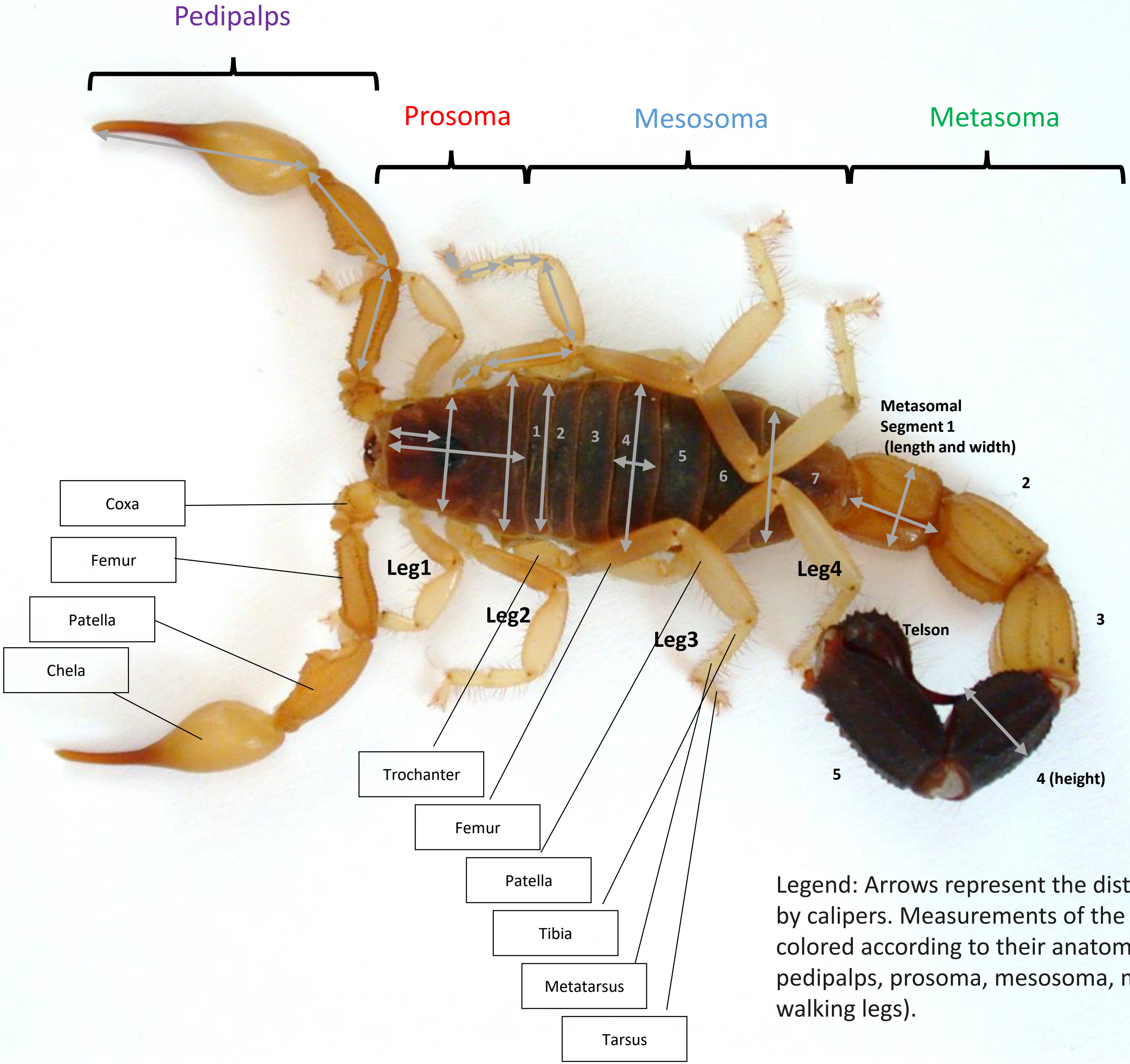

Legend: Arrows represent the distance measured by calipers. Measurements of the left column are colored according to their anatomical region: pedipalps, prosoma, mesosoma, metasoma and walking legs).
