# Supplementary material for: Reevaluating scorpion ecomorphs using a naïve approach
Source: BMC Ecol Evol. 2022 Feb 14;22:17. doi: 10.1186/s12862-022-01968-0 (PMC8845257; doi:10.1186/s12862-022-01968-0)
Supplement: Supplementary file 2 — Additional file 2. Model-Based Clustering performance for clustering of samples via the BIC for up to 10 components (i.e. clusters) (BIC = Bayesian Information Criterion). EII - VVI refer to the names of the models used for clustering fitting. [file 12862_2022_1968_MOESM2_ESM.pdf]

Supplementary Figure 2: Model-Based Clustering performance

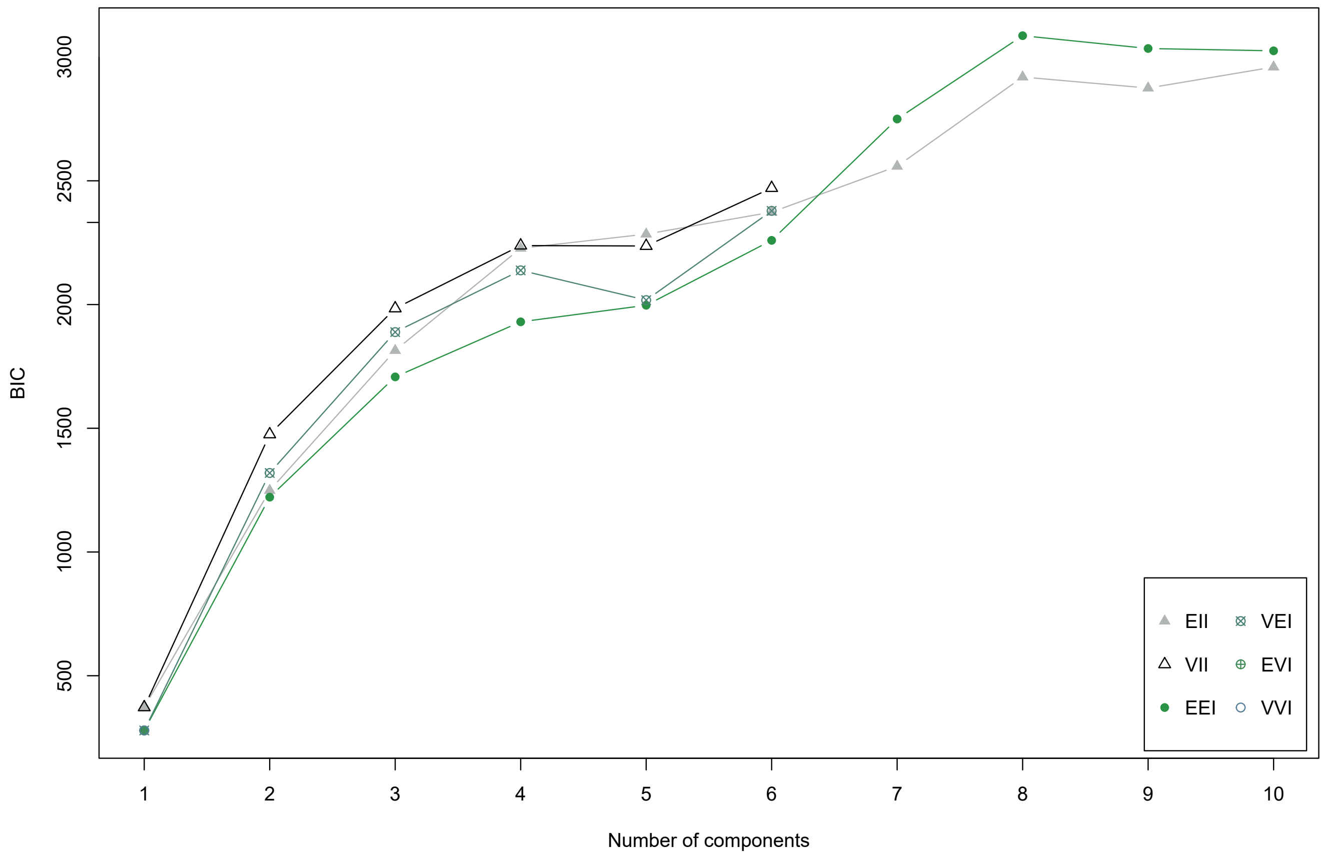

Legend: Model-Based Clustering performance for clustering of samples via the BIC for up to 10 components (i.e. clusters) (BIC = Bayesian Information Criterion). EII - VVI refer to the names of the models used for clustering fitting.
