# Supplementary material for: Reevaluating scorpion ecomorphs using a naïve approach
Source: BMC Ecol Evol. 2022 Feb 14;22:17. doi: 10.1186/s12862-022-01968-0 (PMC8845257; doi:10.1186/s12862-022-01968-0)
Supplement: Supplementary file 3 — Additional file 3. The ecospace is depicted by species (circles) and microhabitat use (triangles) and ecomorph groups (squares) colored according to ecomorph affiliation. The intensity of the colors corresponds to the cos2 correlation with both dimensions. Percentage values refer to the variation explained by each axis. Ecomorph datapoints are supplementary information that does not contribute to axis composition. [file 12862_2022_1968_MOESM3_ESM.pdf]

Supplementary Figure 3. PCoA map of scorpion ecospace.

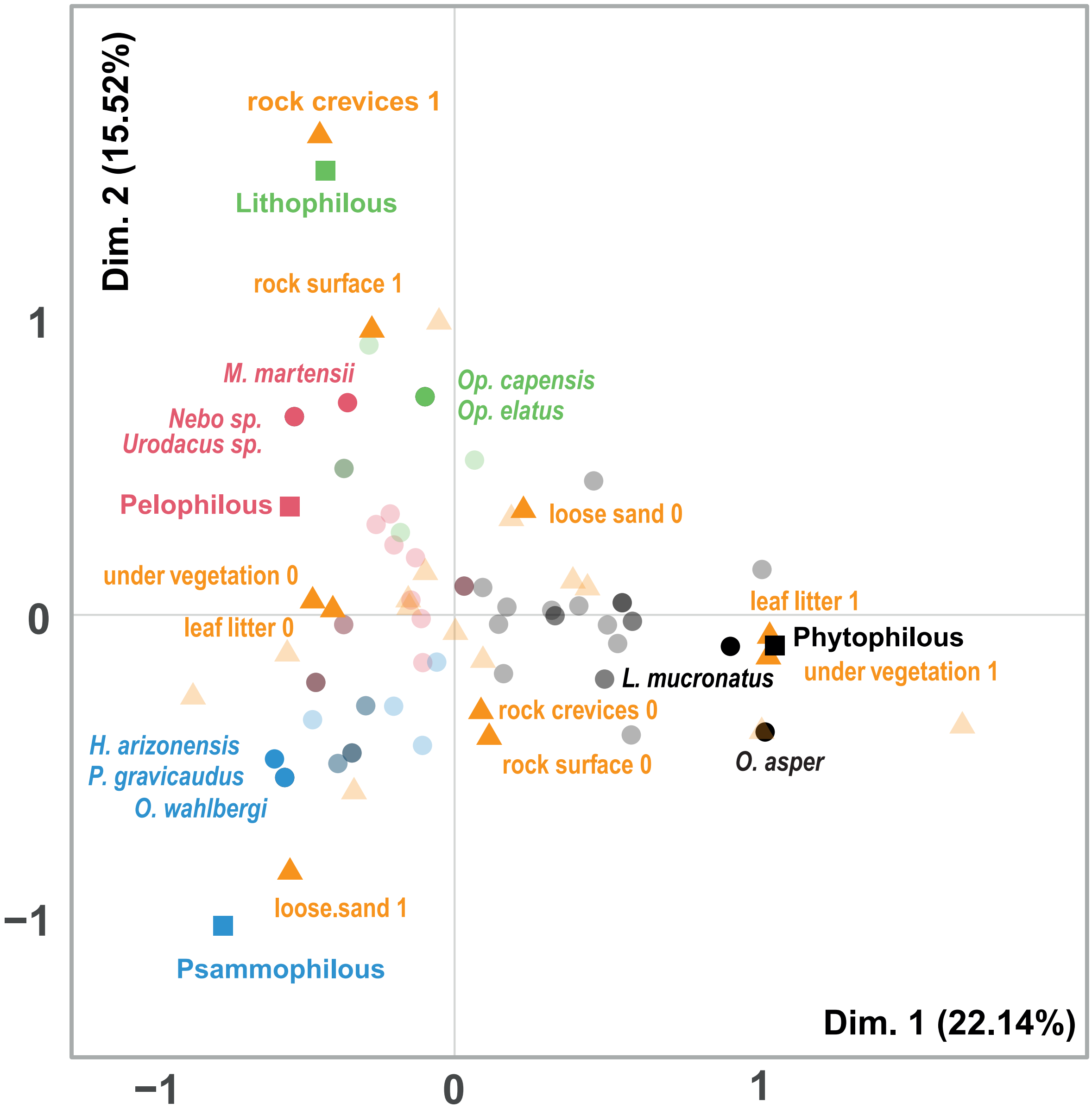

Legend: The ecospace is depicted by species (circles) and microhabitat use (triangles) and ecomorph groups (squares) colored according to ecomorph affiliation. The intensity of the colors correspond to the cos2 correlation with both dimensions. Percentage values refer to the variation explained by each axis. Ecomorph datapoints are supplementary information which do not contribute to axis composition.
