# Supplementary material for: Reevaluating scorpion ecomorphs using a naïve approach
Source: BMC Ecol Evol. 2022 Feb 14;22:17. doi: 10.1186/s12862-022-01968-0 (PMC8845257; doi:10.1186/s12862-022-01968-0)
Supplement: Supplementary file 6 — Additional file 6. Pairwise distances between Least Squares Regression (LSR) means calculated by a MANOVA of eco-projected morphology. [file 12862_2022_1968_MOESM6_ESM.docx]

Supplementary table 3. Pairwise distances between Least Squares Regression (LSR) means calculated by a MANOVA of eco-projected morphology.

| **Pairwise differences** | **d.obs** | **UCL (95%)** | **Z (d)** | **P value** |  |
| --- | --- | --- | --- | --- | --- |
| Lithophilous: Pelophilous | 2.815 | 2.260 | 2.356 | **0.008** |  |
| Lithophilous: Phytophilous | 1.905 | 2.122 | 1.317 | 0.097 |  |
| Lithophilous: Psammophilous | 1.776 | 2.618 | 0.465 | 0.325 |  |
| Pelophilous: Phytophilous | 1.240 | 1.247 | 1.633 | 0.052 |  |
| Pelophilous: Psammophilous | 1.762 | 1.598 | 1.959 | **0.024** |  |
| Phytophilous: Psammophilous | 1.190 | 1.772 | 0.447 | 0.332 |  |

Legend: LSR mean difference (d.obs) with 95 % confidence intervals (UCL), effect sizes (Z), and probabilities of exceeding observed values (P-value) based on 10,000 random permutations. Significant effects at an alpha of 0.05 are marked in bold font.
